# Fully quantitative cardiovascular magnetic resonance myocardial perfusion ready for clinical use: a comparison between cardiovascular magnetic resonance imaging and positron emission tomography

**DOI:** 10.1186/s12968-017-0388-9

**Published:** 2017-10-19

**Authors:** Henrik Engblom, Hui Xue, Shahnaz Akil, Marcus Carlsson, Cecilia Hindorf, Jenny Oddstig, Fredrik Hedeer, Michael S. Hansen, Anthony H. Aletras, Peter Kellman, Håkan Arheden

**Affiliations:** 1 Department of Clinical Physiology, Clinical Sciences, Lund University and Lund University Hospital, Lund, Sweden; 2 National Heart, Lung, and Blood Institute, National Institutes of Health, DHHS, 10 Center Drive, Bethesda, MD 20892 USA; 3 Department of Radiation Physics, Lund University Hospital, Lund, Sweden; 4 Laboratory of Computing, Medical Informatics and Biomedical – Imaging Technologies, School of Medicine, Aristotle University of Thessaloniki, Thessaloniki, Greece

**Keywords:** Myocardial perfusion, Cardiac magnetic resonance, Positron emission tomography, Myocardial perfusion reserve

## Abstract

**Background:**

Recent studies have shown that quantification of myocardial perfusion (MP) at stress and myocardial perfusion reserve (MPR) offer additional diagnostic and prognostic information compared to qualitative and semi-quantitative assessment of myocardial perfusion distribution in patients with coronary artery disease (CAD). Technical advancements have enabled fully automatic quantification of MP using cardiovascular magnetic resonance (CMR) to be performed in-line in a clinical workflow. The aim of this study was to validate the use of the automated CMR perfusion mapping technique for quantification of MP using 13N–NH3 cardiac positron emission tomography (PET) as the reference method.

**Methods:**

Twenty-one patients with stable CAD were included in the study. All patients underwent adenosine stress and rest perfusion imaging with 13N–NH3 PET and a dual sequence, single contrast bolus CMR on the same day. Global and regional MP were quantified both at stress and rest using PET and CMR.

**Results:**

There was good agreement between global MP quantified by PET and CMR both at stress (−0.1 ± 0.5 ml/min/g) and at rest (0 ± 0.2 ml/min/g) with a strong correlation (*r* = 0.92, *p* < 0.001; y = 0.94× + 0.14). Furthermore, there was strong correlation between CMR and PET with regards to regional MP (*r* = 0.83, *p* < 0.001; y = 0.87× + 0.26) with a good agreement (−0.1 ± 0.6 ml/min/g). There was also a significant correlation between CMR and PET with regard to global and regional MPR (*r* = 0.69, *p* = 0.001 and *r* = 0.57, *p* < 0.001, respectively).

**Conclusions:**

There is good agreement between MP quantified by 13N–NH3 PET and dual sequence, single contrast bolus CMR in patients with stable CAD. Thus, CMR is viable in clinical practice for quantification of MP.

## Background

Ischemic heart disease (IHD) is the most common cause of mortality and morbidity in the western world. Obstructive coronary artery disease (CAD) compromising myocardial perfusion is the main pathophysiological mechanism underlying stress-induced myocardial ischemia. The extent of stress-induced ischemia due to CAD has been shown to be of important prognostic value [1, 2]. Significant advances in both diagnosing and treating epicardial CAD have improved survival and reduced morbidity during the last decades in patients suffering from this disease [3–5]. Most clinical methods used for diagnosing stress-induced myocardial ischemia focus on epicardial obstructive CAD and rely on qualitative or semi-quantitative assessment of the relative spatial perfusion distribution i.e. myocardial perfusion single photon emission computed tomography (SPECT), first-pass perfusion cardiac magnetic resonance (CMR) and non-dynamic cardiac positron emission tomography (PET). There are, however, data suggesting that the use of qualitative or semi-quantitative methods for assessing stress-induced ischemia have limited ability to depict the full extent of the atherosclerotic coronary disease [6]. Furthermore, recent studies have shown that full quantification of myocardial perfusion (MP) expressed in terms of ml/min/g myocardial tissue offers independent prognostic information in patients with CAD [7–9].

Cardiovascular magnetic resonance (CMR) with a dual sequence, single contrast bolus approach has been shown to enable quantitative assessment of MP [10]. However, there have been challenges associated with this technique such as the non-linear relationship between signal intensity and gadolinium concentration, respiratory motion correction and gadolinium kinetic modelling. Recent technical developments have shown promising results to overcome these challenges [11]. The proposed CMR perfusion mapping method is implemented in-line, is fully automatic and is readily integrated into the clinical workflow via the Gadgetron framework [12]. Thus, this technique seems to be ready for clinical implementation, making quantitative perfusion available as a routine examination in patients with suspected or established ischemic heart disease. Even though this is a promising technique, clinical validation against an independent reference standard is still lacking.

Cardiac positron emission tomography (PET) with dynamic image acquisition during bolus injection of a radioactive perfusion tracer at rest and stress is currently considered to be the clinical reference method for quantitative assessment of MP. However, cardiac PET is of limited use as a wide spread routine clinical examination due to its limited availability and sometimes challenging logistics as well as due to the ionizing radiation associated with this technique.

Therefore, the aim of this study was to validate MP by a fully automatic, in-line, dual sequence, single contrast bolus CMR perfusion mapping sequence using cardiac 13N–NH3 PET as the reference method in stable CAD patients.

## Methods

### Study population and study design

The study protocol was approved by the regional ethical committee and all subjects gave written informed consent prior to participation. Patients eligible for inclusion had all been clinically evaluated and a decision to perform an elective coronary angiography with preparedness for percutaneous coronary intervention (PCI) had been made, with or without prior stress imaging. Patients were excluded if they had contraindications for CMR such as pacemaker or other CMR-incompatible devices or claustrophobia. The patients were recruited between September 2016 and February 2017 and examined at Lund University Hospital, Lund, Sweden. All patients underwent both adenosine stress/rest CMR with dual sequence, single contrast bolus approach as well as adenosine stress/rest 13N–NH3 PET on the same day (4–5 h apart) for assessment of MP. Rate pressure products (heart rate x systolic blood pressure) were calculated both at rest and stress as a measure of the hemodynamic response to adenosine during PET and CMR, respectively. For 12 patients the CMR and PET examination was performed within a month prior to the angiography. For 9 patients, the CMR and PET examinations were performed as a follow-up approximately 6 months after the angiography.

### Dual sequence, single contrast bolus CMR for MP quantification

#### Image acquisition

All images were acquired on a Magnetom Aera 1.5 T system (Siemens Healthcare, Erlangen, Germany). A basal, a mid-ventricular and an apical short-axis image were acquired both at rest and at stress. The stress images were acquired during 90 heart beats, starting three minutes after initiation of intravenous adenosine infusion (140 μg/kg/min). The arterial input function was calculated using the left ventricular (LV) blood pool which was automatically segmented from low resolution images optimized for the high gadolinium concentration. Higher spatial resolution images were used for estimating myocardial perfusion. In order to achieve a linear relationship between the LV blood signal used as an input function and the gadolinium contrast agent concentration a number of steps were taken in the design of the sequence protocol and image reconstruction as previously described [11]. The sequence uses a low flip angle FLASH low resolution protocol with 2 echoes such that the echo times were short to minimize T2* losses at high concentration, and so that remaining T2* losses could be estimated and corrected. The non-linearity of saturation recovery was minimized by using a short saturation delay achieved using a small matrix and parallel imaging to reduce the number of phase encode lines. The remaining non-linear response is corrected by converting to gadolinium concentration units.

Late gadolinium enhancement (LGE) imaging was performed to assess regions of myocardial infarction.

#### Image reconstruction & perfusion mapping

The input function and myocardial images are converted to units of gadolinium contrast agent concentration (mmol/L) which were input to the tissue model used in quantification [11]. Conversion to gadolinium units effectively addresses the non-linearity between the measured saturation recovery signal and actual gadolinium concentration. Conversion to contrast concentration was accomplished by means of a look-up-table that is calculated by a Bloch simulation of the specific protocol. This look-up-table is recalculated for each scan as part of the image reconstruction. The input to the look-up-table is the measured saturation recovery signal which is normalized by a proton density used as a reference. MP was calculated using a blood tissue exchange model originally developed by Bassingthwaighte [13], which is a distributed model described by partial differential equations. In addition to myocardial blood flow, the model estimates the permeability surface area product for the capillaries which determines the extraction fraction and the intracapillary plasma and interstitial fluid volumes.

#### Image analysis

All images were analyzed using the software Segment (v2.0 R5378) [14]. The endo- and epicardial borders for the basal, mid-ventricular and apical short-axis images were manually delineated both at stress and rest. To avoid inclusion of blood pool or extracardiac structures within the regions of interest where MP was quantified, the delineations were kept approximately 1 pixel away from the endo- and epicardial borders. Obvious image artifacts and coronary arteries were excluded from the regions of interest. Myocardial perfusion in ml/min/g was assessed regionally according to the 17-segment model [15], excluding the apical segment, resulting in 16 segments per patient. MP was assessed globally (average MP for all 16 segments). Myocardial perfusion reserve (MPR) was also calculated, defined as the ratio between MP at stress over rest and was expressed both regionally and globally. Both regional and global MP were compared to corresponding measures by PET. Infarct size was assessed from the LGE short-axis images by the use of the recently described EWA (expectation maximization weighted intensity A priori information*)* algorithm for infarct quantification [16]. In short, the EWA algorithm is based on combining an intensity classification by Expectation Maximization with a pixel intensity weighting approach to account for partial volume effects. Furthermore, a priori information on coronary artery supply territory is taken into consideration for the automatic infarct size quantification.

### Cardiac 13NH_3_-PET for MP quantification

#### Image acquisition and reconstruction

All patients underwent a standard clinical cardiac 13N–NH3 PET at rest and stress, with a minimum of 50 min between the examinations to allow for physical decay of the radiopharmaceutical. All images were acquired with a Discovery 690 PET/CT scanner (General Electric Healthcare, Waukesha, Wisconsin, USA). A CT localization image over the chest was acquired to enable accurate patient positioning, followed by a low-dose computed tomography (CT) used for attenuation correction (120 kV; 10 mAs, 10; rotation time 0.5 s). Thereafter, the patients received an intravenous injection of 430–600 MBq of 13N–NH3 at rest. An ECG-gated PET acquisition with a total duration of 15 min was started simultaneously with the tracer injection. For the stress image acquisition, the patient was injected with the same amount of 13N–NH3 after 3 min of adenosine infusion (140 μg/kg/min) which continued, with continuous ECG monitoring, for 4 min after the injection of the radiopharmaceutical during which the images for quantitative perfusion were acquired.

The 0-4 min PET acquisition data were reconstructed into dynamic images, with 21 time frames (12x5s, 4x10s, 4x20s and 1x60s). The images were reconstructed according to recommendations from the vendor, with OSEM without time-of-flight (TOF), and with point spread function (PSF) modelling, with 3 iterations, 12 subsets and a 5 mm post filter. Static images were reconstructed from the data acquired 4–15 min after start of injection to depict the relative spatial myocardial perfusion using the following reconstruction parameters: OSEM with TOF and PSF modelling with 5 iterations and 18 subsets and a 3 mm post filter. The PET and the CT images were checked for patient motion before reconstruction.

#### Analysis of the quantitative myocardial perfusion

All PET images were analyzed using the software Carimas (version 2.7, Turku, Finland), developed for quantitative perfusion assessment by cardiac PET, including 13N–NH3 [17, 18]. The LV endo-and epicardial borders were automatically segmented with manual adjustment when needed. The arterial input function was derived from a blood pool volume of interest which was placed in the basal part of the LV cavity. The activity in the blood and the myocardial wall as a function of time served as input function for the deGrado compartment model for 13N–NH3 [19] implemented in the Carimas correcting the input function for extraction fraction of 13N–NH3 (approximately 90%). Myocardial perfusion was calculated at rest and stress, both globally and in each of the corresponding 16 segments of the left ventricle used for CMR.

### Statistics

Data are expressed as mean ± standard deviation (SD). The relationship between MP by PET and CMR, both global and regional, as well as MPR was assessed with Pearson’s correlation coefficient. The agreement between the MP by PET and CMR was expressed as bias ± SD and graphically shown as difference plots according to Bland and Altman [20]. The difference in rate pressure products between PET and CMR was assessed by Student’s t-test. All statistical analyses were performed using Excel 2013 (Microsoft Corporation, Redmond, Washington, USA). Results with a *p* value < 0.05 was considered to be statistically significant.

## Results

**Fig. 1:**
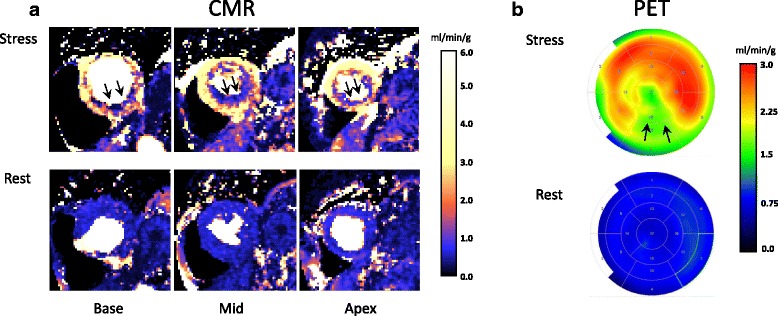
A patient with stress-induced ischemia in the inferior LV wall with a significant stenosis in the right coronary artery. **a** Rest and stress perfusion maps in basal, mid-ventricular and apical short-axis image of the left ventricle acquired using the dual sequence, single contrast bolus CMR perfusion mapping approach, showing stress-induced ischemia in the inferior LV wall (arrows). **b** Rest and stress polar plot PET perfusion maps obtained by dynamic 13N–NH3 imaging showing stress-induced ischemia in the inferior LV wall (arrows) corresponding well with the CMR findings. The central part of the polar plot represents the LV apex and the periphery represents the basal parts of the LV. Of note, the colors for PET and CMR perfusion maps are different and in concordance with colors typically used for each method. Furthermore, the color scales in this case are different for the two methods to optimize visualization of regional pathology. CMR = cardiovascularmagnetic resonance, LV = left ventricle/left ventricular, MP = myocardial perfusion, PET = positron emission tomography

Patient characteristics are shown in Table 1. Typical images obtained by PET and CMR in a patient with right coronary artery (RCA) disease and stress-induced ischemia in the inferior LV wall are shown in Fig. 1. Myocardial infarction was detected in 4 of 21 patients. Infarct size in these 4 patients was 8 ± 5%. Of the 12 patients that underwent CMR and PET before angiography, 4 patients had signs of stenosis in left anterior descending coronary artery (LAD) of whom 3 underwent revascularization, 3 patients had stenosis in RCA of whom 2 underwent revascularization, 1 patient had stenosis in the left circumflex coronary artery (LCX) who underwent revascularization and 6 patients had no significant stenosis. Of the patients with CMR and PET 6 months after angiography 4 patients had stenosis in LAD of whom 3 had been revascularized, 2 had stenosis in RCA of whom 1 had been revascularized, 2 patients had stenosis in LCX of whom 1 had been revascularized and 2 patients had no significant stenosis. Complete records of the PET and CMR rate pressure products at both rest and stress were available for 12 patients. There were similar mean rate pressure products for PET and CMR both at rest (8932 ± 1563 vs 9189 ± 1756, *p* = 0.37) and at stress (10,945 ± 2170 vs 11,351 ± 2463, *p* = 0.53). Furthermore, the difference between stress and rest rate pressure products did not differ between PET and CMR (2014 ± 2554 vs 2162 ± 2120, *p* = 0.75) indicating similar hemodynamic responses to adenosine stress.

**Table 1:** Patient characteristics

Age (years)	68 ± 9
Female	4 (19%)
Smoking	3 (14%)
Prior CABG	0 (0%)
Prior PCI	5 (24%)
Prior MI	3 (14%)
Diabetes	4 (19%)
Hypertension	14 (67%)
Hyperlipidemia	12 (57%)
MI by LGE	4 (19%)
Beta-blockers	12 (60%)
Statins	20 (95%)
ACE-inhibitors/ARB	9 (43%)
Anti-coagulants	17 (81%)

*ACE* angiotensin converting enzyme, *ARB* angiotensin receptor blocker, *CABG* coronary artery bypass grafting, *LGE* late gadolinium enhancement, *MI* myocardial infarction, *PCI* percutaneous coronary intervention

### Global MP

**Fig. 2:**
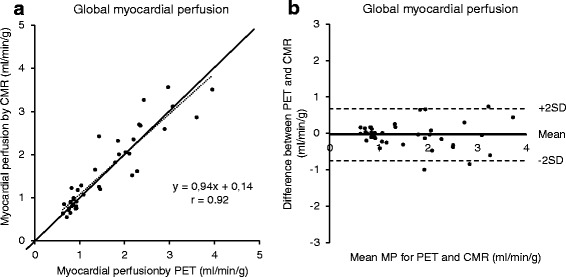
The relationship between PET and CMR for global myocardial perfusion rest and stress included, **a**). The dashed line represents the line of regression. The solid line indicates the line of identity. **b** Bland-Altman plots for the difference between PET and CMR for global MP. The solid line represents the mean difference between the two methods and the dashed lines indicate +2SD and -2SD, respectively. There is strong correlation and small bias between PET and CMR for assessing global MP. CMR = cardiovascular magnetic resonance, MP = myocardial perfusion, PET = positron emission tomography

The global MP at rest and stress ranged from 0.6–4.0 ml/min/g and 0.6–3.8 ml/min/g for PET and CMR, respectively. Figure 2a shows that there was a strong correlation (*r* = 0.92 *p* < 0.001; y = 0.94× + 0.14) between MP quantified by PET and CMR. There was a good agreement between PET and CMR both at stress (−0.1 ± 0.5 ml/min/g) and at rest (0 ± 0.2 ml/min/g) (Fig. 2b). Furthermore, there was a significant correlation between global MPR by PET and CMR (*r* = 0.69, *p* = 0.001; y = 0.83× + 0.53) and a good agreement (−0.1 ± 0.6 ml/min/g).

### Regional MP

**Fig. 3:**
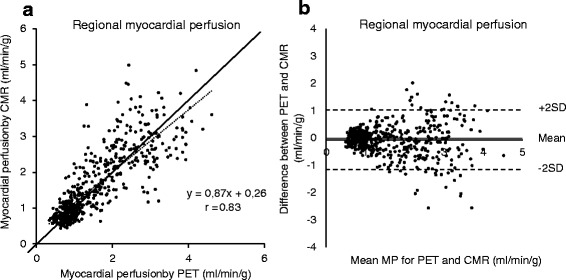
The relationship between PET and CMR for regional myocardial perfusion (rest and stress included, **a**). The dashed line represents the line of regression. The solid line indicates the line of identity. **b** Bland-Altman plots for the difference between PET and CMR for regional MP. The solid line represents the mean difference between the two methods. The dashed lines indicate +2SD and -2SD, respectively. There is significant correlation and small bias between PET and CMR for assessing regional MP in 16 of the 17 segments of the left ventricle. CMR = cardiovascular magnetic resonance, MP = myocardial perfusion, PET = positron emission tomography

The regional MP at rest and stress ranged from 0.4–4.6 ml/min/g and 0.5–5.3 ml/min/g for PET and CMR, respectively. Figure 3a shows the correlation between regional MP by PET and CMR (*r* = 0.83, *p* < 0.001; y =  0.87× + 0.26). There was a good agreement between PET and CMR both at stress (−0.1 ± 0.7 ml/min/g) and at rest (0 ± 0.3 ml/min/g) (Fig. 3b). The correlation between MPR by PET and CMR was *r* = 0.57, *p* < 0.001 (y = 0.68× + 1.02) with a good agreement (0 ± 1.1 ml/min/g). Table 2 shows what the angiographer evaluated as significant stenosis by angiography in relation to regional stress MP < 2.0 ml/min/g as recently suggested as optimal cut-off for 13N–NH3 PET for detection of significant coronary stenosis [21].

**Table 2:** Comparison between angiography and regional myocardial stress perfusion < 2.0 ml/min/g for the 12 patients with PET (A) and CMR (B) before angiography

A		Angiography		B		Angiography	
		+	–			+	–
PET	+	6	3	CMR	+	4	2
	–	0	3		–	2	4

### Global MP reduction

**Fig. 4:**
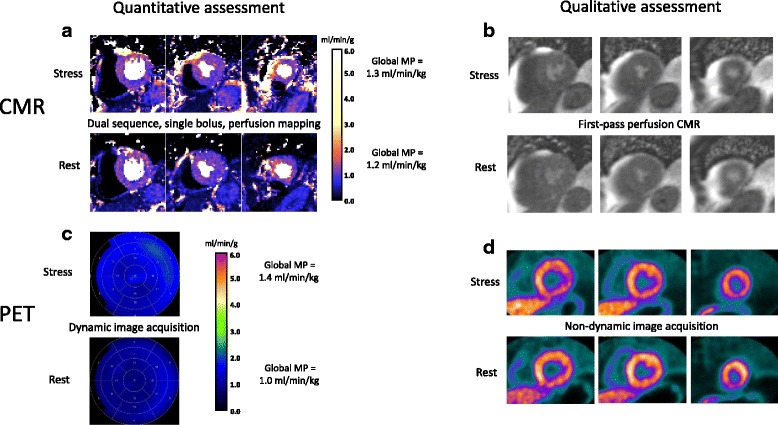
A patient with multi-vessel disease. **a** CMR MP maps at rest and stress, **b** first-pass perfusion images at rest and stress, **c** PET polar plot MP maps from dynamic 13 N–NH3 PET acquisition at rest and stress and **d** non-dynamic 13 N–NH3 PET images at rest and stress. Note the absence of any regional perfusion defect in any of (**a**-**d**). For the quantitative assessment of MP (**a**, **c**), the patient had signs of a global decrease in myocardial perfusion reserve with only a slight increase of MP during stress as assessed both with PET and CMR. However, for the qualitative assessment of relative perfusion distribution (**b**, **d**), the patient was evaluated as having normal distribution and no stress-induced ischemia. The angiography for this patient showed signs of stenosis in all three main coronary arteries. CMR = cardiovascular magnetic resonance, MP = myocardial perfusion, PET = positron emission tomography

Absolute quantification of MP is particularly important in cases exhibiting a global reduction in stress flow which arises in multi-vessel disease or microvascular disease. Figure 4 shows an example of a patient with a deceased global MP without regional ischemia on qualitative assessment of perfusion distribution. The MP appears to be normal in the raw first-pass CMR perfusion images (Fig. 4b) and in the non-dynamic, non-quantitative PET images (Fig. 4d), used in routine clinical practice for CMR and nuclear cardiology, respectively. Absolute quantification of MP (Fig. 4 a,c), however, clearly shows a significant reduction in flow and corresponding low flow reserve which could be attributed to either microvascular disease or multi-vessel disease. In this case, multi-vessel disease was confirmed by invasive angiography.

## Discussion

The present study shows that fully quantitative CMR MP mapping is ready for clinical use by validating the method against 13N–NH3 PET, which was used as the independent reference standard. There was strong correlation between the two methods with negligible bias for both global and regional MP. Thus, using CMR for quantifying MP is an attractive alternative to PET in the clinic. However, routine clinical implementation of quantitative CMR MP will rely on its importance on therapeutic decision making, which needs to be proven by large clinical outcome trials that use MP as an endpoint.

Quantification of MP and MPR in routine clinical practice will have important implications on the management of patients with established or suspected CAD. Even though a normal perfusion scan with qualitative or semi-quantitative evaluation of first-pass perfusion has been shown to predict low risk of future coronary events [22–24], there remain patients in this group with adverse prognosis due to multi-vessel disease or non-obstructive CAD [25–27]. Thus, even in the absence of obstructive CAD, such as for 3 of 12 patients in Table 2, patients with pathologic MP at stress and low MPR are most likely in need of medical attention. The pathophysiological mechanisms explaining a decreased MP in the absence of obstructive coronary artery disease are likely related to disease states shown to affect the coronary microvascular function, such as dyslipidemia [28, 29], hypertension [30], diabetes [31, 32], and smoking [33]. Significant vascular dysfunction related to these diseases may precede development of obstructive CAD [34], which makes quantification of MP and MPR potential diagnostic methods for identifying patients at risk of developing coronary stenosis. Furthermore, there are currently patients being treated with PCI in coronaries supplying myocardium with normal MPR [35]. Thus, these patients are put at risk associated with invasive treatment (endothelial injury, bleeding complications, risk of coronary artery dissection etc.) with little or no benefit to be gained from the procedure.

Using relative perfusion distribution, typically applied in routine clinical practice of nuclear cardiology, for diagnosing ischemic heart disease means that the counts in the perfusion maps are normalized to the highest regional counts. Thus, there might be variability in counts within normal myocardium due to variability of perfusion within the normal range. Hence, parts of the normal myocardium might have lower counts, potentially interpreted as ischemia and therefore compromising the diagnostic accuracy as recently shown by Kajander et al. [36]. They showed that the positive and negative predictive values for detecting or excluding invasively diagnosed fractional flow reserve (FFR)-positive coronary stenoses decreased significantly when using relative spatial perfusion distribution instead of quantitative MP.

One major challenge with quantitative perfusion imaging, including both CMR and PET in the present study, is to find a perfusion tracer with uptake kinetics that reflects the actual myocardial perfusion over a wide range of blood flows. It has previously been shown that 13N–NH3 uptake is approximately linearly proportional to flow at low MPs but that there is an underestimation of actual blood flow at high flows, which was compensated for by correcting for ammonia extraction [37]. The same underestimation has been shown for CMR and gadolinium-based extracellular contrast agents [38]. In the current approach [11], however, the gadolinium extraction fraction is estimated and used in calculating MP to avoid this underestimation of MP for CMR.

Imaging for both PET and CMR was initiated at the same time point (3 min) following the start of adenosine infusion. However, the duration for PET imaging and adenosine administration for the PET examination was significantly longer than for CMR. Thus, the adenosine response may differ somewhat [39] affecting the validation to some extent.

The results in the present study indicate that a dual sequence, single contrast bolus CMR perfusion imaging and 13N–NH3 PET have similar abilities in terms of quantifying myocardial blood flow over a wide range of flows. Thus, these techniques should be interchangeable and could therefore be used in accordance with local experience and expertise on the method of choice. A major limitation with 13N–NH3 PET is the cumbersome logistics with producing the tracer, requiring a cyclotron close to the imaging facility. Given the short half-life of 13N–NH3 (< 10 min) the tracer has to be produced and transported to the PET scanner within a limited time window. This requires advanced logistics and significant manpower, which also affects the cost per examination. With dual sequence, single contrast bolus CMR there are no such limitations. CMR offers superior characterization of myocardial morphology and function compared to 13N–NH3 PET due to its excellent spatial resolution. Furthermore, PET imaging is associated with ionizing radiation which CMR is not, making CMR the method of choice in younger patients and in situations where serial follow-up examinations is expected to be necessary, such as monitoring of treatment effects.

For wide spread clinical use of quantification of CMR-based MP, image acquisition, reconstruction and post-processing need to be both automated and standardized. The CMR technique described in the present study is highly automated and standardized with regards to imaging protocol, image reconstruction and post-processing of the data. The same requirements regarding automation and standardization is true for cardiac PET involving different perfusion tracers and different mathematical data modeling for quantification of MP built into different imaging software. The use of 13N–NH3 for quantification of MP and MPR has recently been shown to be stable across different software implementations [40].

Quantification requires knowledge of the amount of contrast agent in the myocardial tissue and the arterial input function (AIF) driving the delivery of this contrast agent. Accurate quantification is challenged by the lack of linearity between the measured signal and contrast agent concentration. The main sources of non-linearity and bias are: spatial signal variations caused by the sensitivity profiles of the surface coils, imperfect saturation of magnetization during contrast bolus passage, T2* decay (and signal loss) caused by high contrast agent concentrations in the blood pool, and the non-linear signal response inherent due to saturation recovery that depends on the parameters of the imaging protocol. The proposed dual sequence [11] is designed to address all of these issues. It has been proposed that some of the non-linearity of the AIF response curve can be mitigated by imaging the AIF during a separate injection of a bolus with lower concentration (the dual bolus approach) [41], but this approach has some practical drawbacks as it requires multiple injections and a longer overall acquisition time. Moreover, there are other potential bias sources with this approach, since changes in breathing, etc. between the two measurements may introduce new sources of variation. Consequently, it is desirable to acquire the AIF curve simultaneously with the tissue response curve.

## Conclusions

There is a good agreement between MP quantified by CMR with inline perfusion flow mapping and 13N–NH3 PET in patients with suspected stable CAD. Thus, CMR is a viable alternative in clinical practice for quantification of MP.

## Abbreviations

ACE: Angiotensin converting enzyme
ARB: Angiotensin receptor blocker
CABG: Coronary artery bypass grafting
CAD: Coronary artery disease
CMR: Cardiovascular magnetic resonance
CT: Computed tomography
ECG: Electrocardiography
EWA: Expectation maximization, weighted intensity A priori information
FFR: Fractional flow reserve
IHD: Ischemic heart disease
LGE: Late gadolinium enhancement
LV: Left ventricle/left ventricular
MI: Myocardial infarction
MP: Myocardial perfusion
MPR: Myocardial perfusion reserve
OSEM: Ordered subsets expectation maximization
PCI: Percutaneous coronary intervention
PET: Positron emission tomography
PSF: Point spread function
SD: Standard deviation
SPECT: Single photon emission computed tomography
TOF: Time-of-flight
